# Sport and exercise habits in children with juvenile idiopathic arthritis (JIA)

**DOI:** 10.1186/1546-0096-9-S1-P126

**Published:** 2011-09-14

**Authors:** M Nørgaard, T Herlin

**Affiliations:** 1 Department of Physiotherapy, Aarhus University Hospital, Skejby, Denmark; 2 Department of Pediatrics, Aarhus University Hospital, Skejby, Denmark

## Background

Over the last decade implementation of targeted therapy has improved the functional ability in JIA-children. Also, the attitude towards participation of JIA-children in sport has become less restrictive with growing evidence of the benefits of physical activity on e.g. disease, fitness and quality of life.

## Aim

To explore the habits in gym classes and sport in 10-16 year-old JIA-children compared to gender- and age-matched healthy controls.

## Methods

68 JIA-children (females 60.3%), age 12.74 (±1.70) years, disease duration 5.72 (±4.17) years, and 118 healthy controls (females 50.8%), age 12.36 (±1.74) years, answered questionnaires on sport and exercise habits and functional ability (CHAQ38). For JIA: VAS-pain, Patient- and Physician-GA were noted.

## Results

Mean CHAQ38 scores for JIA: 0.1896 (±0.2025), controls: 0.0359 (±0.0799) indicated only minimal functional impairment in both groups. Pain assessments correlated well to CHAQ38 scores in JIA (p-values <0.01).

No significant differences were found between sport-active JIA-children and controls concerning type and number of sport activities or amount of time spent in sport. Significantly more JIA-children were not sport-active (38% (26/68) vs. 25% (29/118)), mainly due to joint pain (12/26) and getting short of breath/side-stitches (9/26).

Significantly fewer JIA-children participated fully in gym (p<0.01) and reported pain (91% (62/68)) and difficulties with certain activities (p<0.01). Strategies mainly used when pain; a short break (81%), changing activity (58%), continuing despite pain (37%).

## Conclusion

JIA-children still participate less in sport activities and are more challenged in gym classes than healthy peers, despite close-to-normal functional ability. However, sport-active JIA-children do not differ significantly from sport-active healthy peers.

